# Supplementary material for: Early establishment and life course stability of sex biases in the human brain transcriptome
Source: Cell Genom. 2025 May 26;5(7):100890. doi: 10.1016/j.xgen.2025.100890 (PMC12278626; doi:10.1016/j.xgen.2025.100890)
Supplement: Document S1. Figures S1–S25 [file mmc1.pdf]

Cell Genomics, Volume *5*

Supplemental information

Early establishment and life course stability of sex biases in the human brain transcriptome

Clara Benoit-Pilven, Juho V. Asteljoki, Jaakko T. Leinonen, Juha Karjalainen, Mark J. Daly, and Taru Tukiainen

Document S1

related to Benoit-Pilven et al. “Early establishment and life course stability of sex biases in the human brain transcriptome”, Cell Genomics

Supplemental Figures

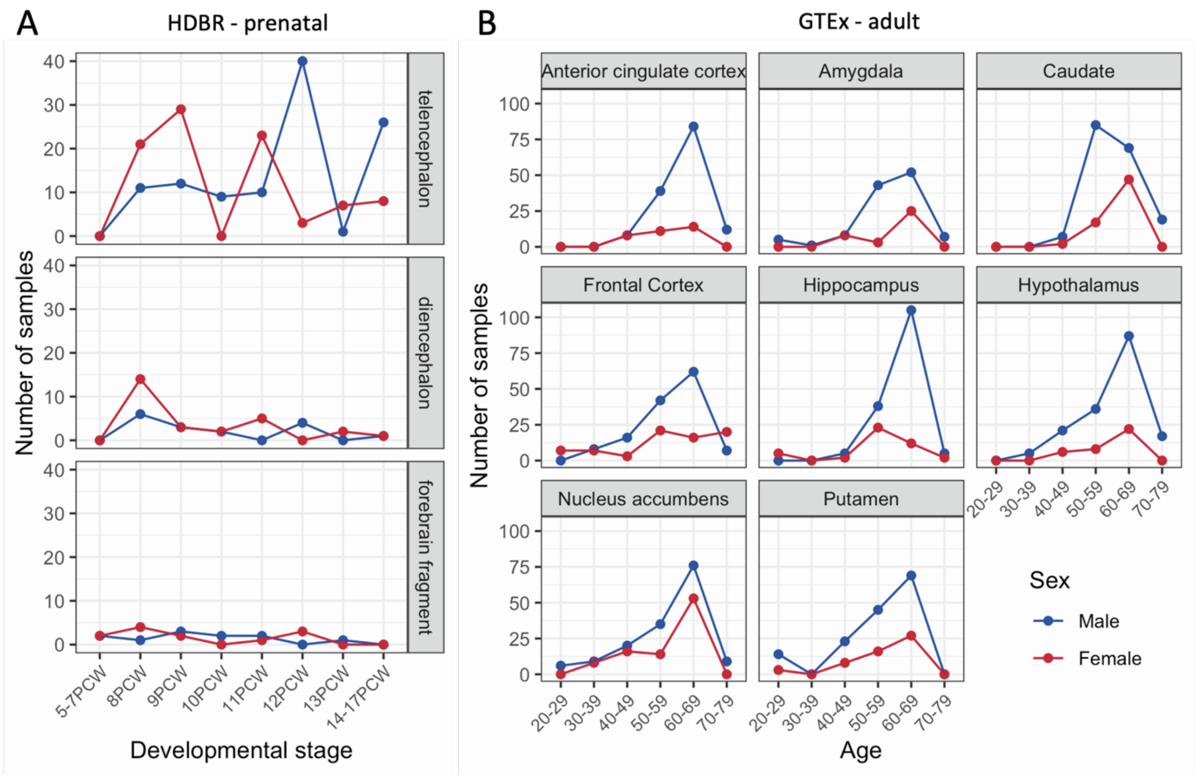

Figure S1: Number of samples per sex, developmental stages/ages and forebrain region, related to Figure 1. A.) Numbers for HDBR. B.) Numbers for GTEx.

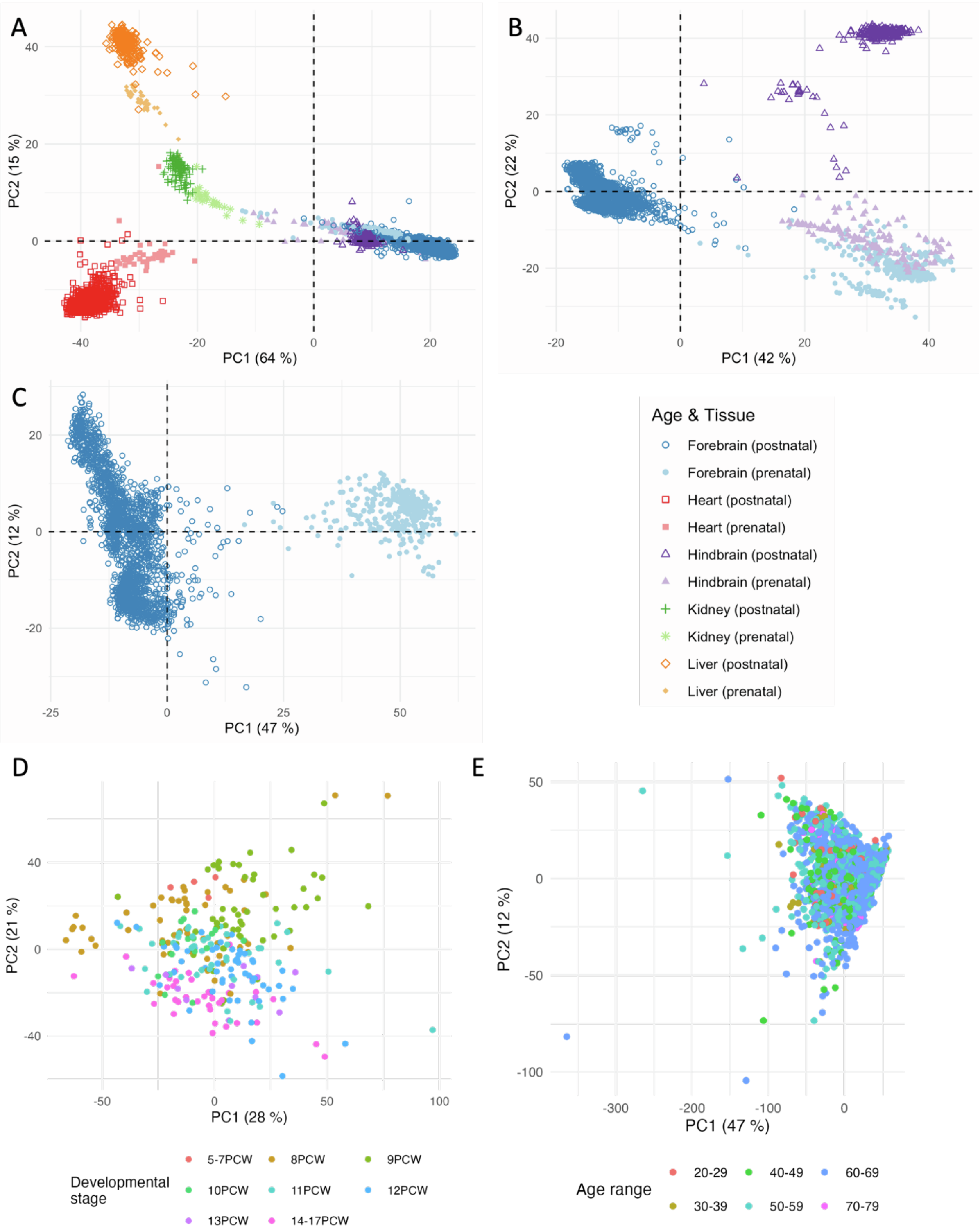

A
PC1 (64 %)
PC2 (15 %)
B
PC1 (42 %)
PC2 (22 %)
C
PC1 (47 %)
PC2 (12 %)
Age & Tissue
Forebrain (postnatal)
Forebrain (prenatal)
Heart (postnatal)
Heart (prenatal)
Hindbrain (postnatal)
Hindbrain (prenatal)
Kidney (postnatal)
Kidney (prenatal)
Liver (postnatal)
Liver (prenatal)
D
PC1 (28 %)
PC2 (21 %)
Developmental stage
5-7PCW
8PCW
9PCW
10PCW
11PCW
12PCW
13PCW
14-17PCW
E
PC1 (47 %)
PC2 (12 %)
Age range
20-29
40-49
60-69
30-39
50-59
70-79

Figure S2: Principal component analysis (PCA) plots for prenatal and postnatal samples, related to Figure 1. A.) PCA plot of all tissues. B.) PCA plot of brain tissues. C.) PCA plot of forebrain samples. Each sample is colored by tissue type and their shape corresponds to the sample age (prenatal and postnatal). D.) PCA plot for prenatal forebrain samples from HDBR colored and shaped by developmental stage. E.) PCA plot for adult forebrain samples from GTEx colored by age. In the PCA with samples from all tissues and life stages (panel A), we observe a clear separation between the different tissues without a clear distinction between pre- and postnatal samples. While the two subregions of the brain cluster together in this analysis, if we analyze only the brain samples, we see that they have a distinct transcriptomic profile in adults (panel B). Finally, focusing only on the forebrain samples, we observe a clear distinction in the transcriptomic profile of the forebrain between the pre- and postnatal life stages (panel C).

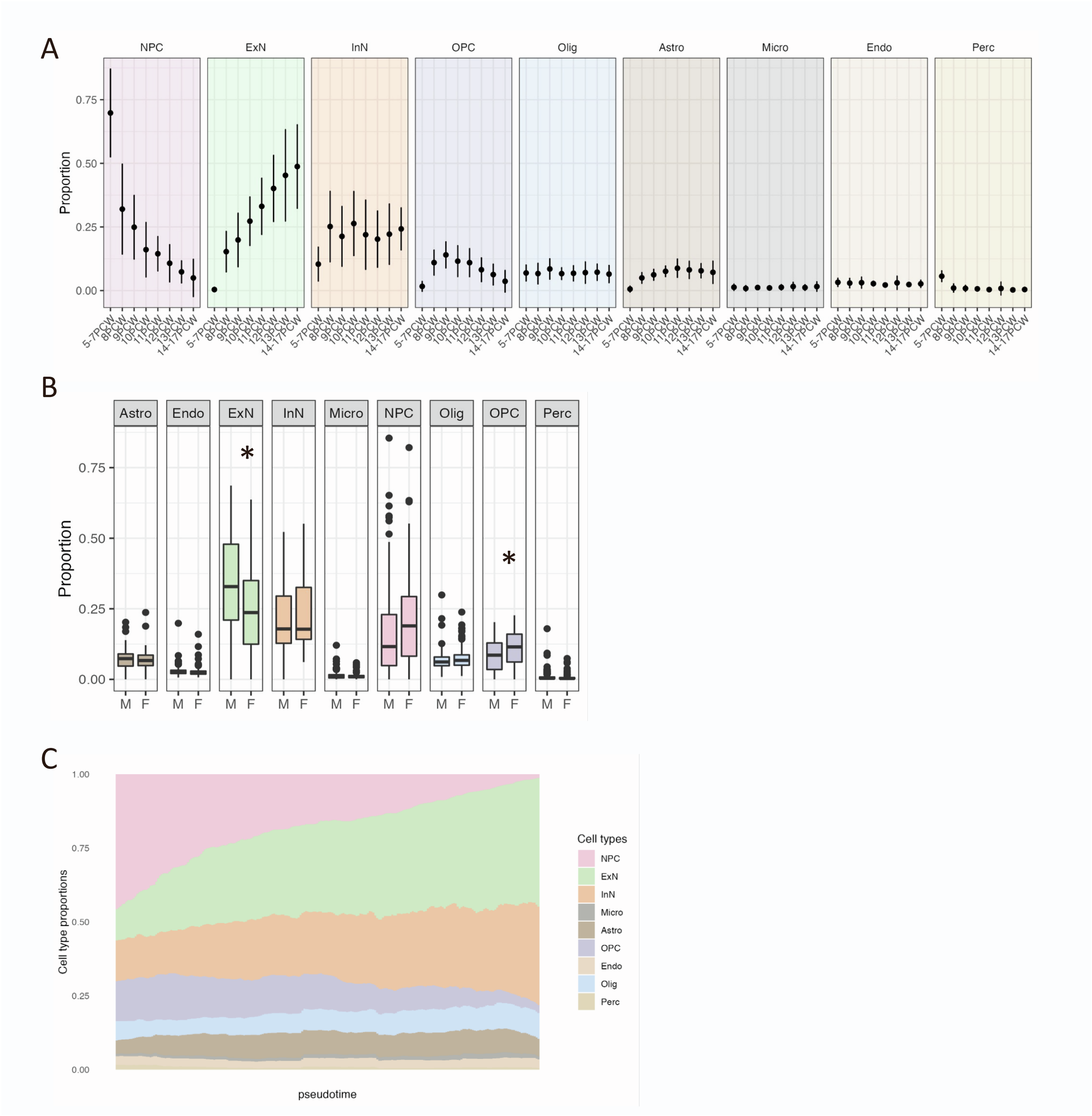

Figure S3: Cell type decomposition in HDBR, related to Figure 1. A.) Proportion and SE of each cell type for the prenatal samples grouped by developmental stage. B.) Cell type proportion split by sample sex. C.) Moving average of cell type composition along pseudotime. *: adjusted p-value < 0.05 from Wilcoxon test. NPC = Neural Progenitor Cells; ExN = Excitatory Neurons; InN = Inhibitory Neurons; OPC = Oligodendrocyte Progenitor Cells; Olig = Oligodendrocytes; Astro = Astrocytes; Micro = Microglial cells; Endo = Endo; Perc = Pericytes.

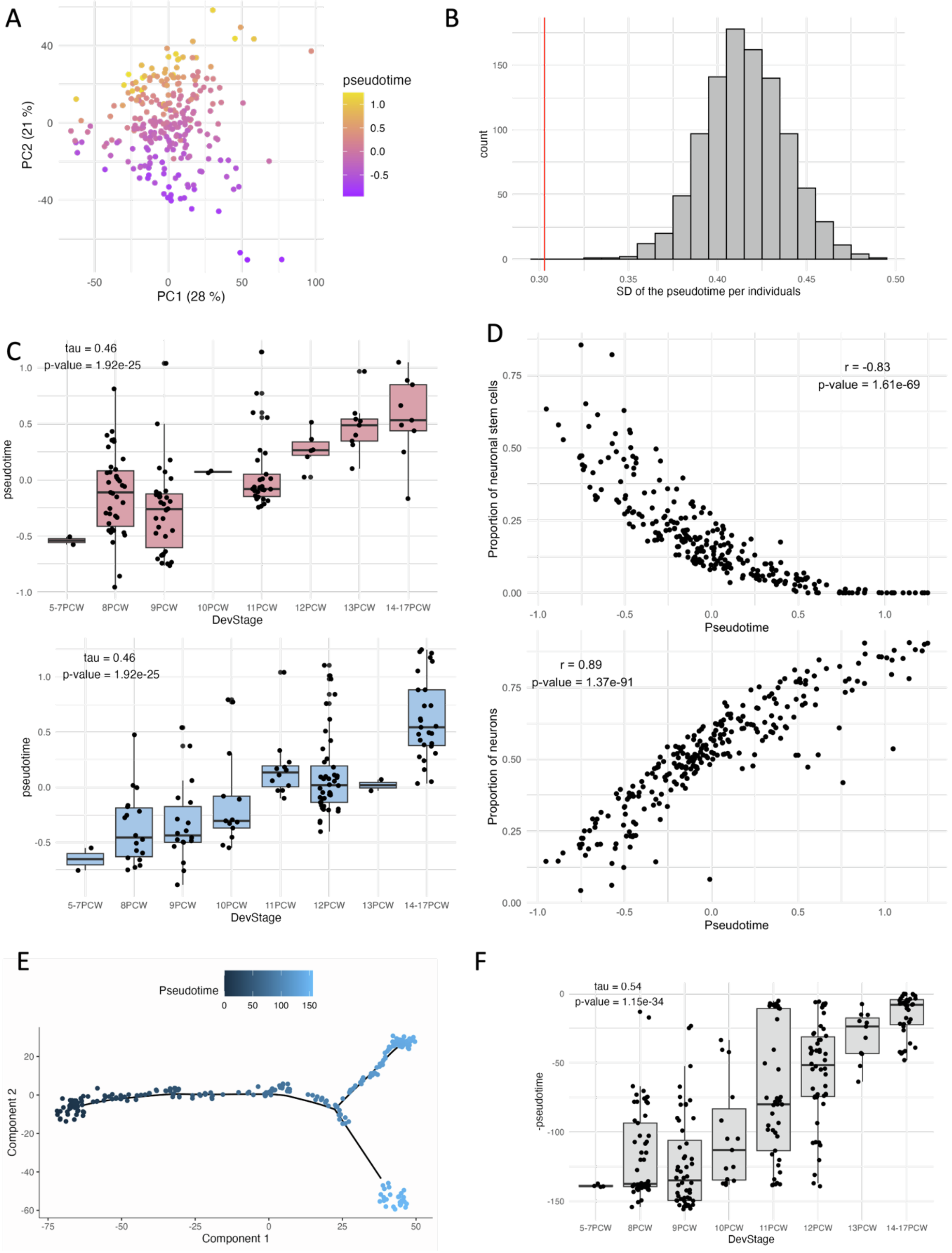
A
PC2 (21 %)
PC1 (28 %)
pseudotime
B
count
SD of the pseudotime per individuals
C
tau = 0.46
p-value = 1.92e-25
pseudotime
DevStage
5-7PCW
8PCW
9PCW
10PCW
11PCW
12PCW
13PCW
14-17PCW
D
Proportion of neuronal stem cells
r = -0.83
p-value = 1.61e-69
Pseudotime
tau = 0.46
p-value = 1.92e-25
pseudotime
DevStage
Proportion of neurons
r = 0.89
p-value = 1.37e-91
Pseudotime
E
Pseudotime
Component 2
Component 1
F
tau = 0.54
p-value = 1.15e-34
-pseudotime
DevStage

Figure S4: Pseudotime analysis on prenatal data, related to Figure 1. A.) PCA plot of the prenatal samples colored by pseudotime. B.) Permutation test (1000 permutations, p-value<0.001) for the mean standard deviation (SD) of samples from the same individual. Mean pseudotime standard deviation (SD) between samples from the same individuals = 0.30 (indicated by the read vertical line) vs mean pseudotime SD between random samples = 0.42. C.) Correlation between pseudotime and developmental stage separated by sample sex. Top: females, bottom: males. D.) Proportions of neuronal stem cells and neurons inferred with CIBERSORTx along pseudotime. E.) Pseudotime analysis with monocle. Inferred sample trajectory colored by pseudotime. F.) Correlation between developmental stage and pseudotime inferred by monocle. Similar correlation with the developmental stages is seen for the Monocle2 inferred pseudotime as for the original phenopath pseudotime.

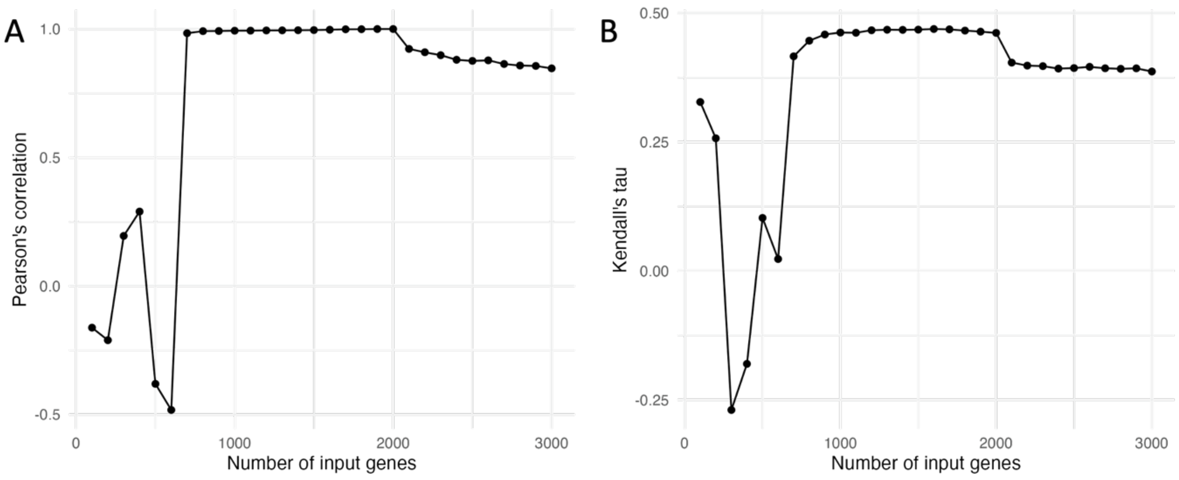

Figure S5: Analysis of the impact of the input genes on the pseudotime inference, related to Figure 1. A.) Pearson's correlation between the original pseudotime and the new inferred pseudotime with the number of input genes ranging from 100 to 3000. B.) Kendall's tau values between the developmental stages and the new inferred pseudotime with the same range of input genes as in A. Reducing the number of input genes to the phenopath algorithm, even down to 700 most variable genes, maintains the estimated pseudotime values very consistent with the original pseudotime inferred using the 2000 most variable genes (Pearson's r>0.98). Accordingly, these pseudotime estimates also show a good correlation with the developmental stages (Kendall's tau>0.41), and across the iterations, the highest correlations are observed between pseudotimes inferred with 1000-2000 input genes. With a larger number of input genes (>2000), the correlations with both the original pseudotime and the developmental stages remain generally high, but are, nevertheless, lower than with the 1000-2000 input genes. A smaller number (<700) of input genes starts to introduce more variability in the pseudotime and also drops correlation with the developmental stages dramatically, suggesting these genes capture a more variable presentation of the samples. Given the drastic change in the correlation of pseudotime and developmental stages between the pseudotime inferred with 601 or 700 most variable genes in the data set, we reasoned that these 100 genes are most informative for understanding the processes captured by the pseudotime metric that reflects the developmental trajectory. Supporting this idea, we find that in GO enrichment analysis these 100 genes are enriched, e.g., in processes related to development and neurogenesis (such as "regulation of nervous system development" or "regulation of neurogenesis").

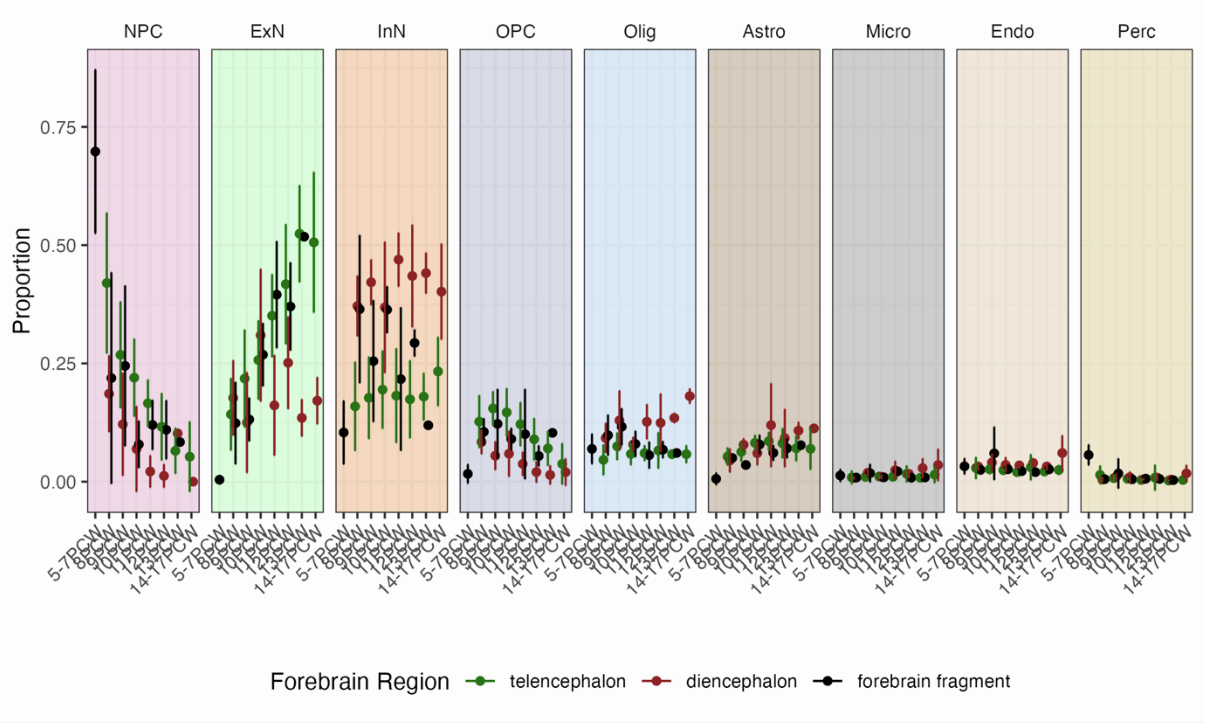

Figure S6: Mean cell-type proportion by developmental stage and forebrain region in prenatal data, related to Figure 1. Each cell-type is shown in its own colored rectangle. The cell-type proportions were estimated with CIBERSORTx for each sample. NPC: neuronal progenitor cells, NPC = Neural Progenitor Cells; ExN = Excitatory Neurons; InN = Inhibitory Neurons; OPC = Oligodendrocyte Progenitor Cells; Olig = Oligodendrocytes; Astro = Astrocytes; Micro = Microglial cells; Endo = Endo; Perc = Pericytes.

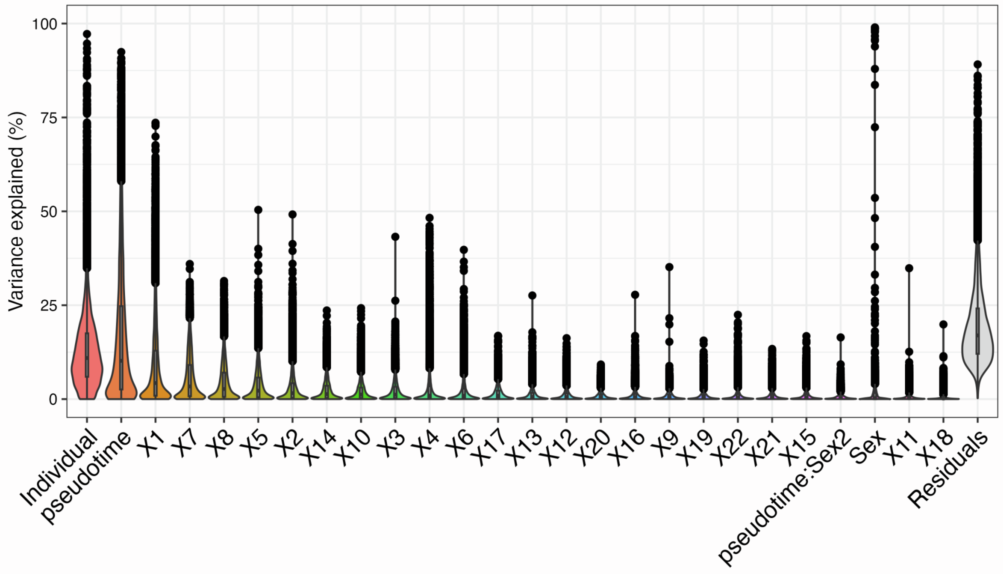

Figure S7: Variance explained by each variable in the model used in the HDBR DE analysis, related to Figure 1.

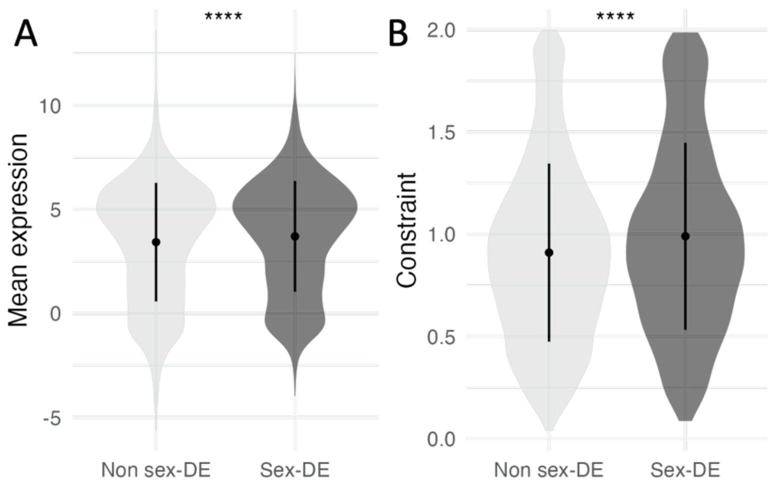

Figure S8: Characteristics for prenatal sex-DE genes (N=3187) compared to non-sex-DE genes (N=14414), related to Figure 1. A.) Comparison of the average gene expression in the prenatal brain between the two groups of genes. B.) Comparison of gene constraint (LOEUF) between the two groups of genes. Smaller LOEUF values indicate higher gene constraint. Wilcoxon test p-value: **** <= 0.0001.

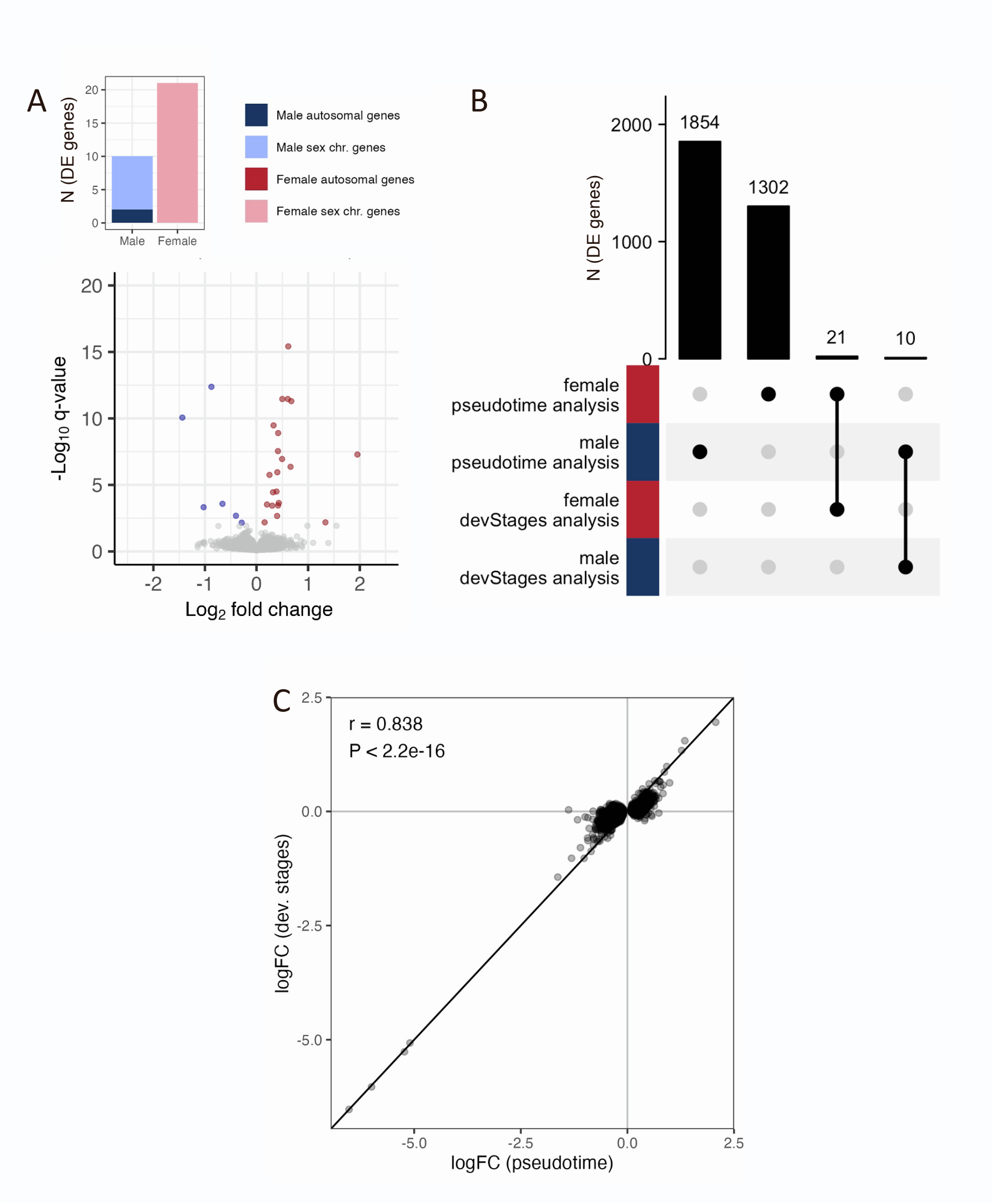

Figure S9: Sex-DE analysis in prenatal dataset with categorical developmental stages as a covariate, related to Figure 1. A.) Barplot of the number of sex-DE genes and volcano plot (female-biased genes are shown in red and male-biased genes in blue). There are only 31 sex-biased genes (q-value<0.01) in the sex-DE analysis with categorical developmental stages, which is in great contrast to the 3187 sex-DE genes identified in the sex-DE analysis pseudotime variable. B.) Comparison of the sex-DE analyses with pseudotime and categorical developmental stages as a covariate with an upset plot of the common significant sex-DE genes. C.) Correlation of the effect sizes (logFC) between the two analyses for genes significantly differentially expressed in the sex-DE analysis using the pseudotime as a covariate. A similar high correlation is detected when only autosomal sex-DE genes are included (Pearson correlation

r=0.790, p-value<1x10-10). There is also a high consistency in sex-DE effect directions (87% consistency for the sex-DE genes from the sex-DE analysis with the pseudotime covariate, permuted p-value<0.001). Further, the π1 statistic indicates that a significant fraction of the sex-DE genes from sex-DE analysis with pseudotime show non-null p-values in sex-DE analysis using the developmental stages (π1=0.42).

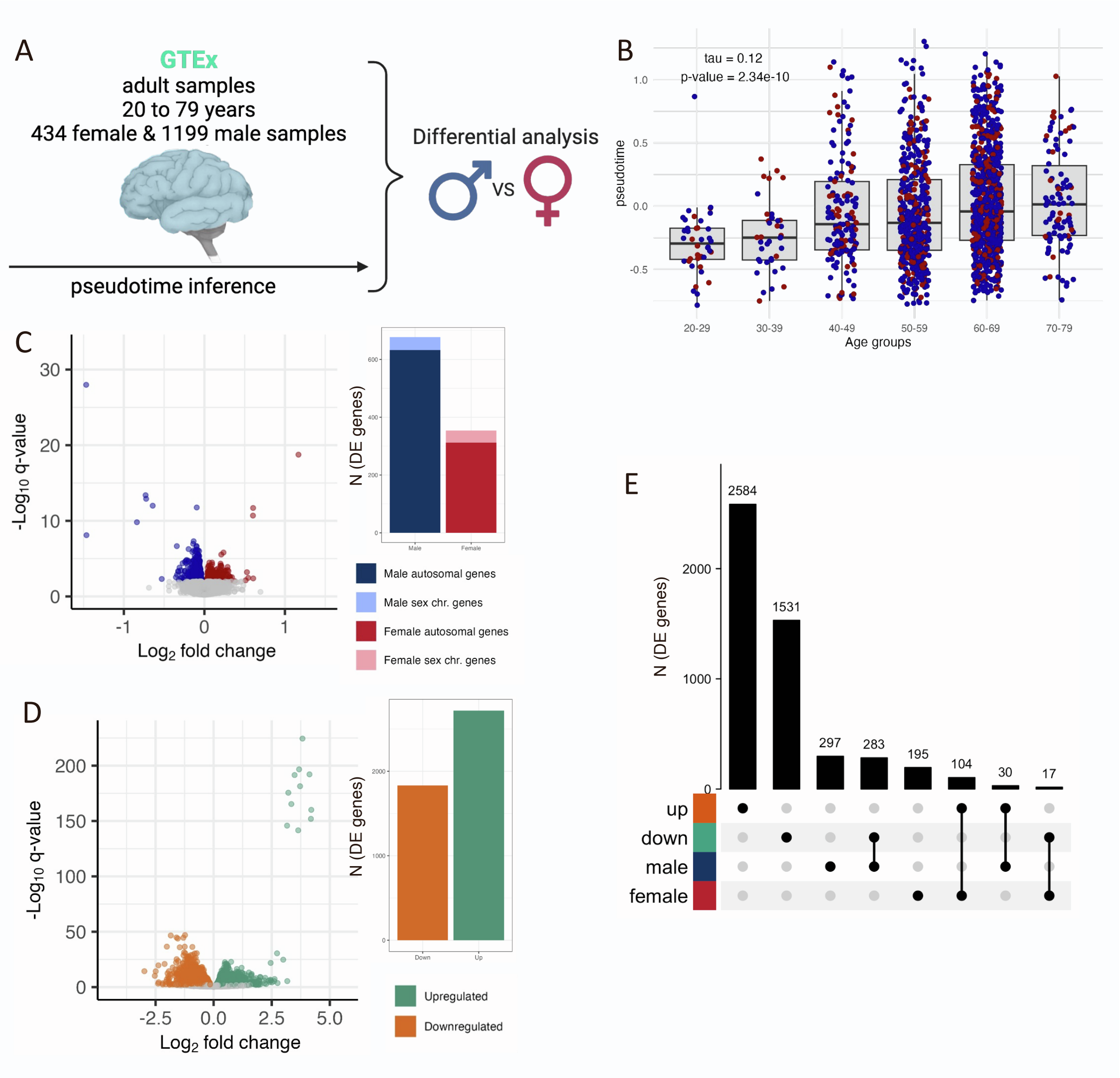

Figure S10: Pseudotime and DE analysis for GTEx forebrain dataset, related to Figure 2. A.) The data set consisted of 1633 adult samples from 337 distinct individuals ranging from 20 to 79 years of age. A pseudotime analysis followed by a differential expression analysis comparing males and females was carried out using this data. B.) Correlation between pseudotime and age groups. C.) Barplot of the number of DE genes (q-value < 0.1 and |log2FC| > 0.01) and volcanoplot for the sex-DE analysis. On the volcanoplot only autosomal genes are shown and only significant genes are colored. D.) Barplot of the number of DE genes (q-value < 0.1 and |log2FC| > 0.01) and volcanoplot for the pseudotime-DE analysis. On the volcanoplot only significant genes are colored. E.) Intersection of the two DE analyses as an upset plot taking into account the direction of effect. The upper barplot shows the size of the intersections.

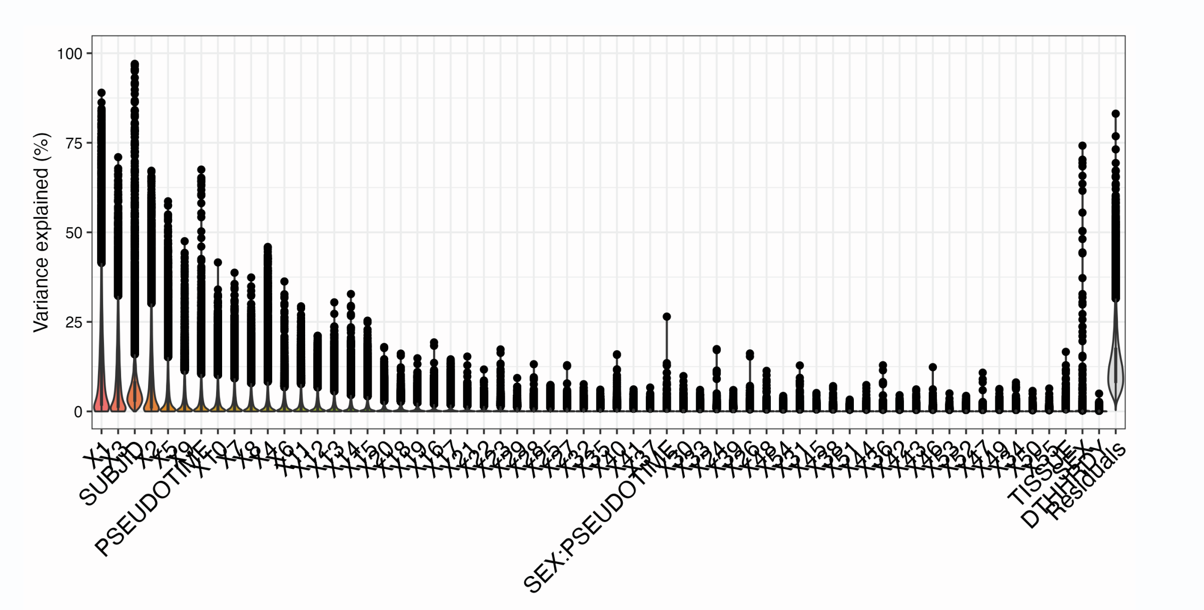

Figure S11: Variance explained by each variable in the model used in the GTEx DE analysis, related to Figure 2.

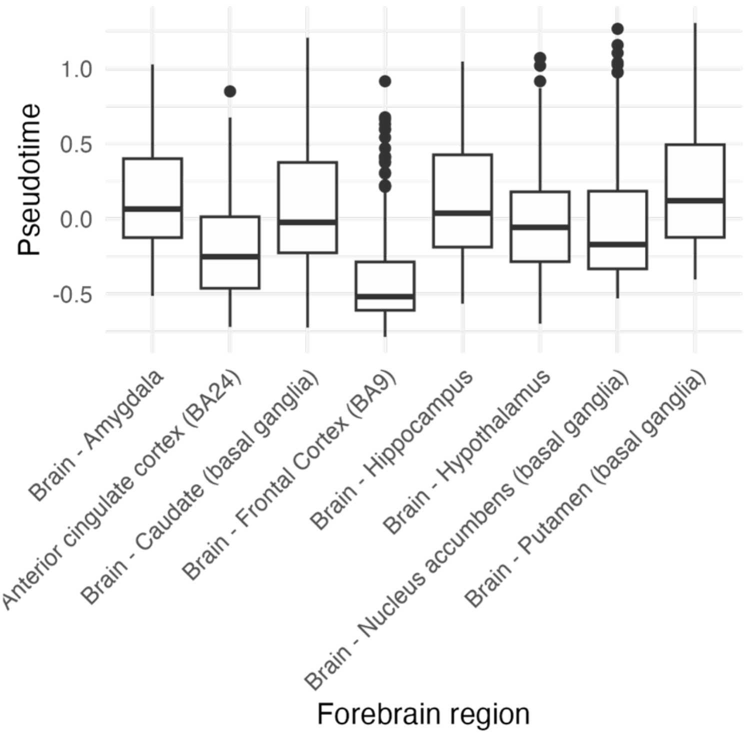

Figure S12: Boxplot of pseudotimes per forebrain region in the adult dataset, related to Figure 2. Some differences in the pseudotime estimates between samples from different brain regions are observed. For instance, samples from the frontal cortex typically received pseudotime estimates smaller than those from the hippocampus within an individual (paired Wilcoxon test p-value=2.8x10-25). This suggests pseudotime can partly reflect cell-type-dependent expression patterns.

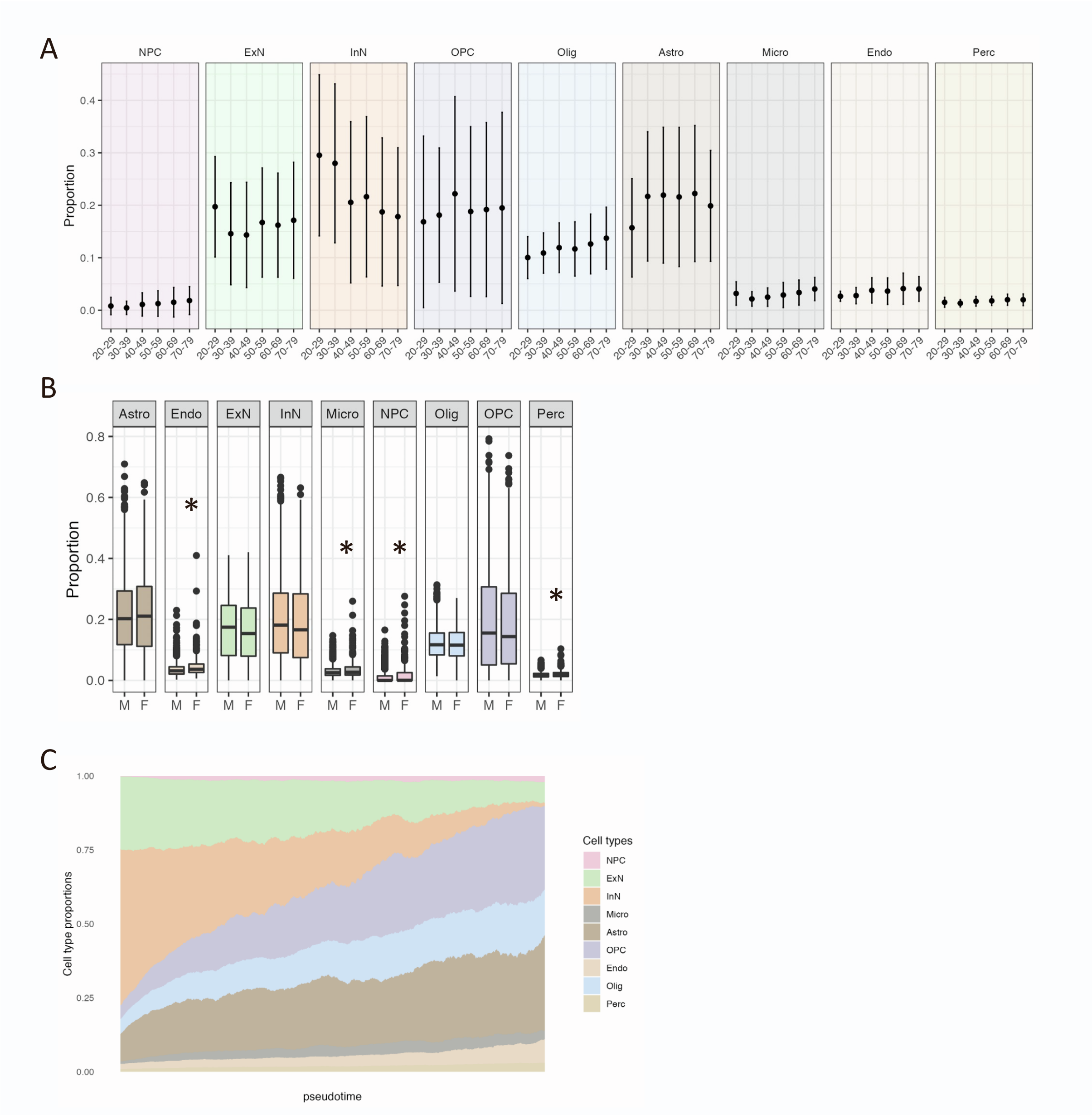

Figure S13: Cell type decomposition in GTEx, related to Figure 2. A.) Proportion and SE of each cell type for the prenatal samples grouped by age category. B.) Cell type proportion split by sample sex. C.) Moving average of cell type composition along pseudotime. *: adjusted p-value < 0.05 from Wilcoxon test. NPC = Neural Progenitor Cells; ExN = Excitatory Neurons; InN = Inhibitory Neurons; OPC = Oligodendrocyte Progenitor Cells; Olig = Oligodendrocytes; Astro = Astrocytes; Micro = Microglial cells; Endo = Endo; Perc = Pericytes.

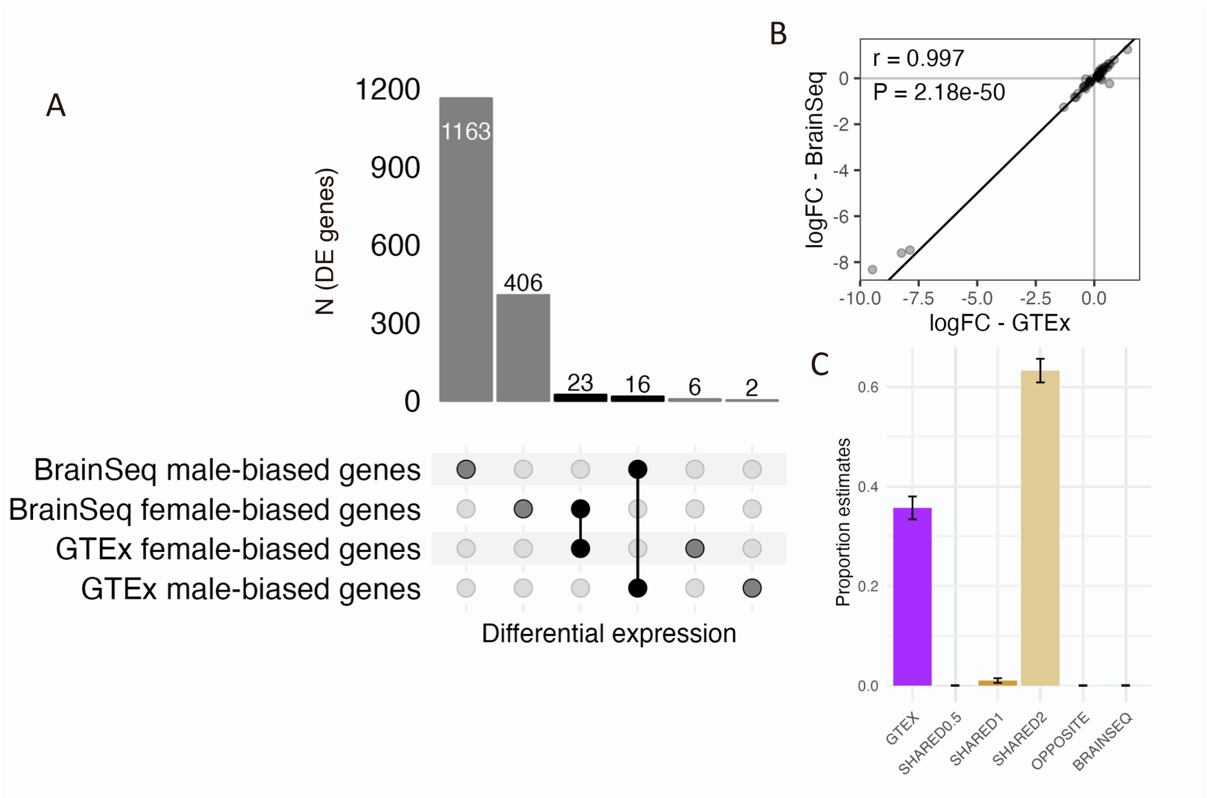

Figure S14: Comparison of sex-DE genes in the same tissue (cortex), related to Figure 2. These comparisons use two independent datasets: GTEx prefrontal cortex (N=209) and BrainSeq phase1 DLPFC (N=189). A.) Upset plot showing the overlap of the DE results from the two datasets accounting for the direction of effect. B.) Correlation of the logFC of the GTEx sex-DE genes between the two datasets. C.) Proportion estimates and SE of each gene category in the Bayesian analysis: GTEx-specific (GTEX), shared with effect size twice larger in GTEx data (SHARED0.5), shared with same effect size (SHARED1), shared with effect size twice larger in BrainSeq brain (SHARED2), opposite effect size and BrainSeq-specific (BRAINSEQ).

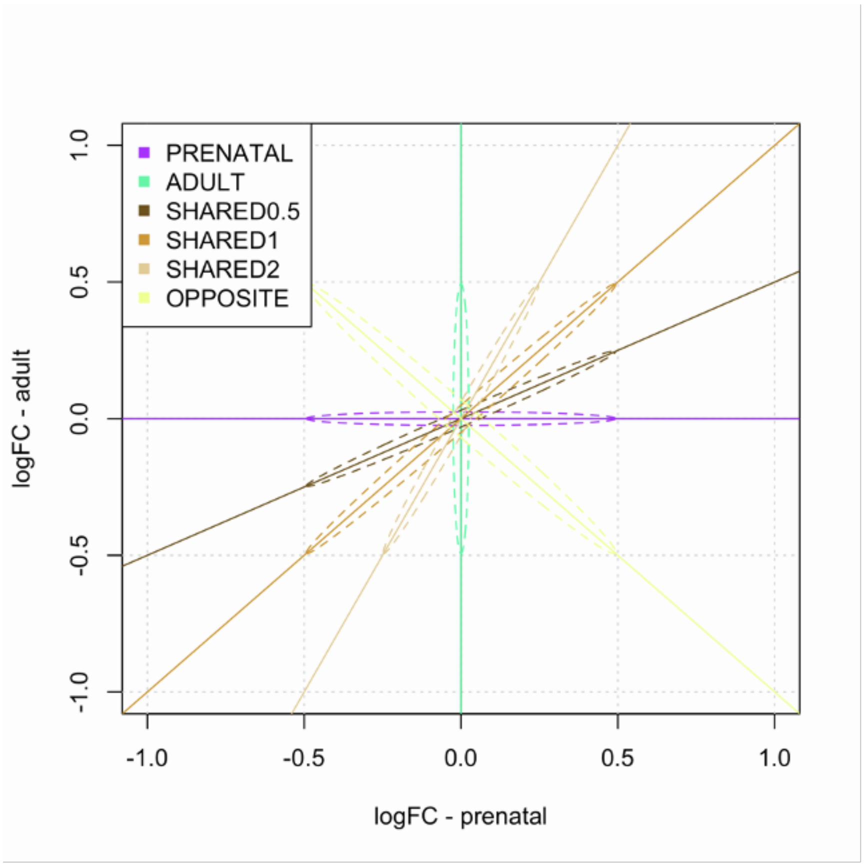

Figure S15: LineModel plot showing the lines of the 6 models used in the Bayesian analysis, related to Figure 2.

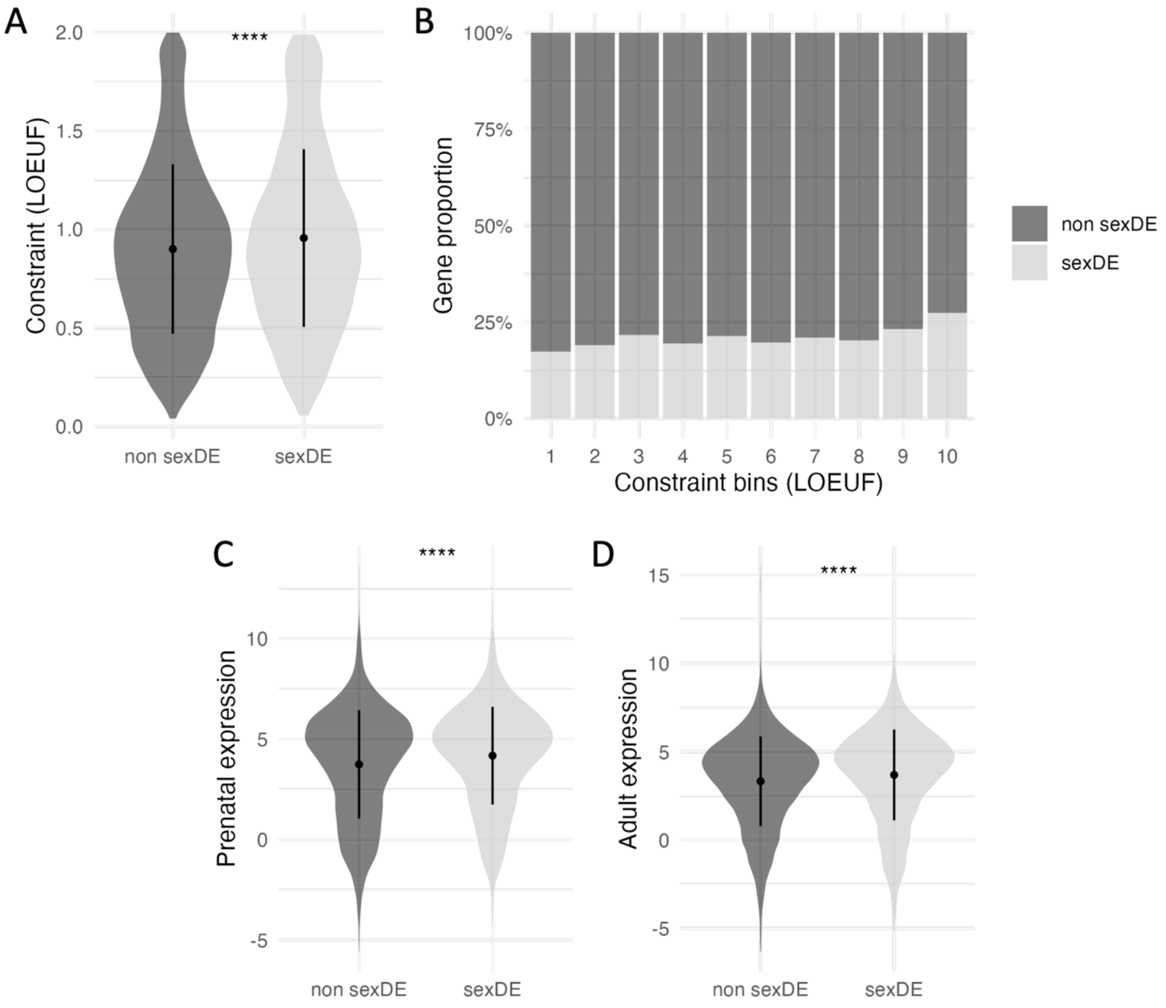

Figure S16: Characteristics of sex-DE genes in either adult or prenatal forebrain (N=3356) compared to non-sex-DE genes, related to Figure 2. A.) Comparison of gene constraint (LOEUF) between the two groups of genes. B.) Proportion of sex-DE and non-sex-DE genes in each bin of gene constraint. The bins correspond to the 10 deciles of the LOEUF score. C.) Comparison of gene expression between the two groups of genes in the prenatal forebrain. D.) Comparison of gene expression between the two groups of genes in the adult forebrain. Wilcoxon test p-value: **** <= 0.0001.

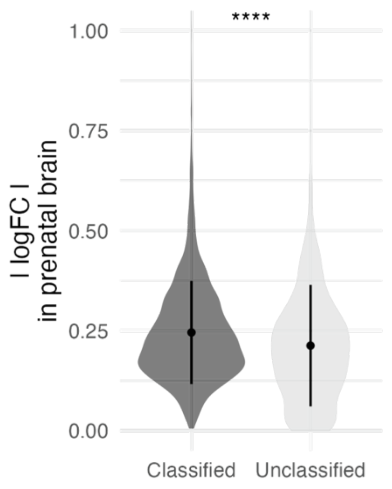

Figure S17: Difference in effect size, |logFC|, in the prenatal brain between the sex-DE genes classified in one of the 6 categories and the unclassified ones, related to Figure 2.

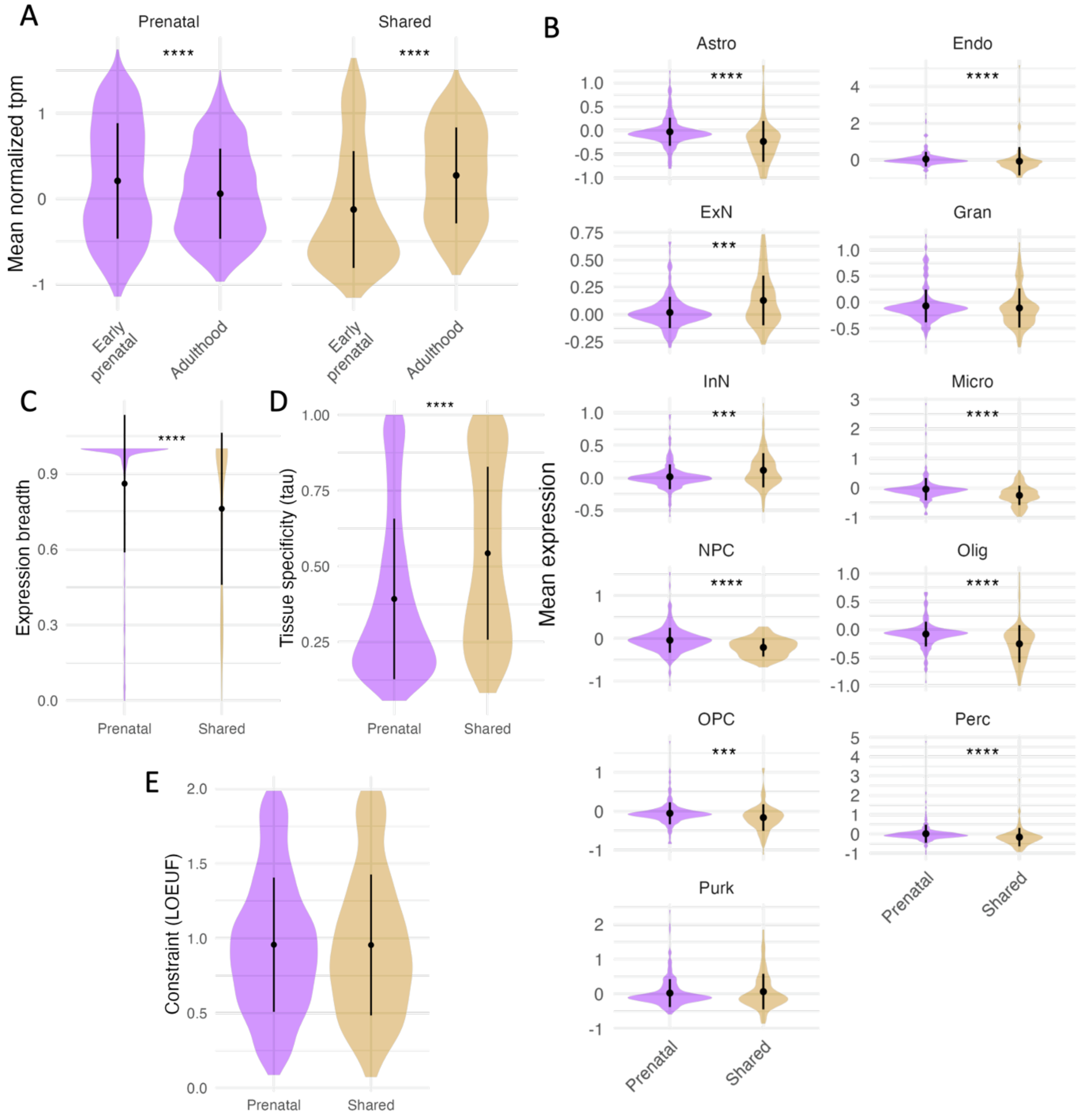

Figure S18: Characteristics of shared and prenatal-specific sex-DE genes between adult and prenatal forebrain, related to Figure 3. A.) Comparison of average gene expression by gene category between BrainSpan early prenatal and adulthood samples. B.) Comparison of average gene expression per gene category in each cell type found in brain single-cell RNA-seq data. C.) Comparison of the expression breadth between the shared and prenatal-specific sex-DE genes. The expression breadth of a gene is defined as the percentage of GTEx tissues in which a gene is expressed (TPM >= 1). D.) Comparison of the tissue specificity (tau value) between the shared and prenatal-specific sex-DE genes. E.) Comparison of the gene constraint

metric, LOEUF, between the shared and prenatal-specific sex-DE genes Wilcoxon test adjusted p-value: **** <=0.0001, *** <= 0.001.

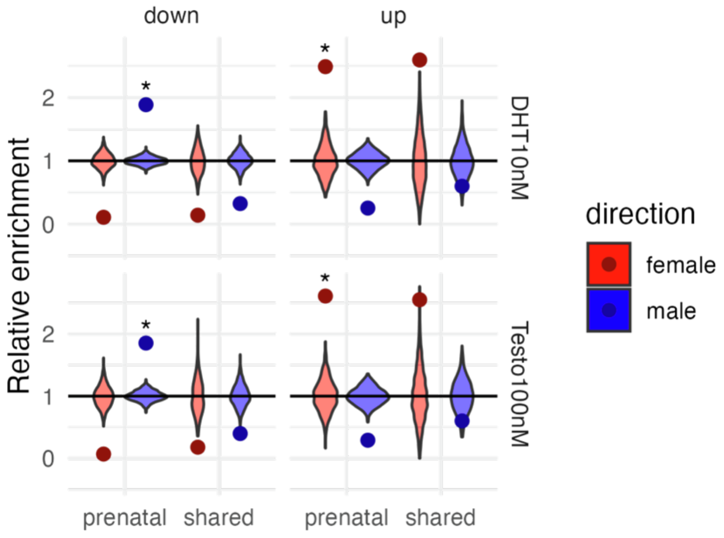

Figure S19: Enrichment analysis of shared and prenatal-specific sex-DE genes with genes regulated by androgen treatment, related to Figure 3. Data from two androgen treatments was analysed: testosterone (Testo 100nM) and dihydrotestosterone (DHT 10nM). The enrichment of the list of genes of interest (dot) is compared to the enrichment of 1000 random gene lists (violinplot). * indicates gene lists passing the significance threshold for both the hypergeometric test (for the list of interest) and the permutation test (compared to the random gene sets), as defined in Methods.

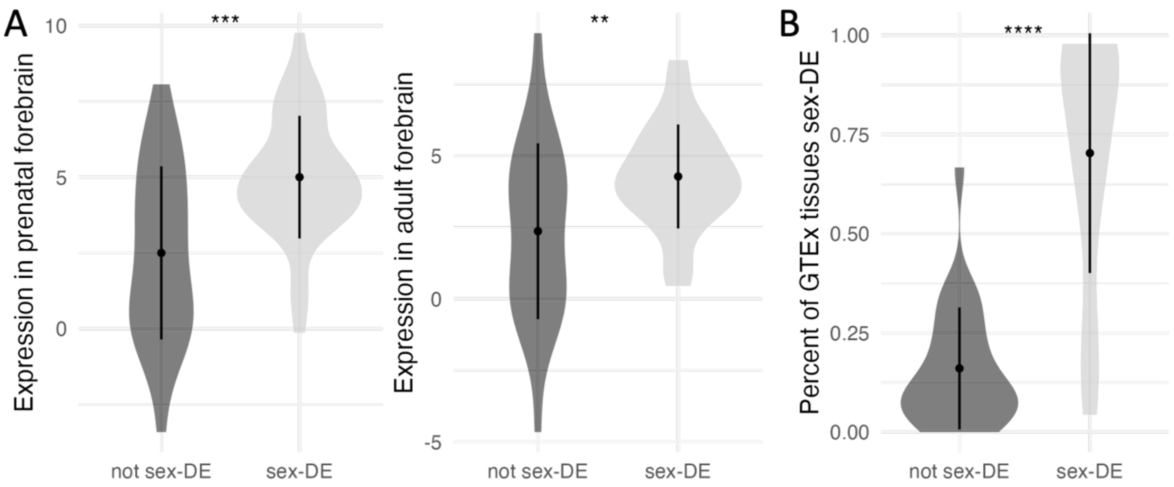

Figure S20: Characteristics of established X-chromosome escape genes found as sex-DE in at least one of the two life-stages (N=42) or in neither of them (N=25), related to Figure 3. A.) Comparison of the average expression of the two groups in either prenatal forebrain (left) or adult forebrain (right). B.) Percentage of GTEx tissues showing sex-DE effect (with q-value < 0.01) for each group of genes. Wilcoxon test p-value: **** <= 0.0001, *** <= 0.001, ** <= 0.01.

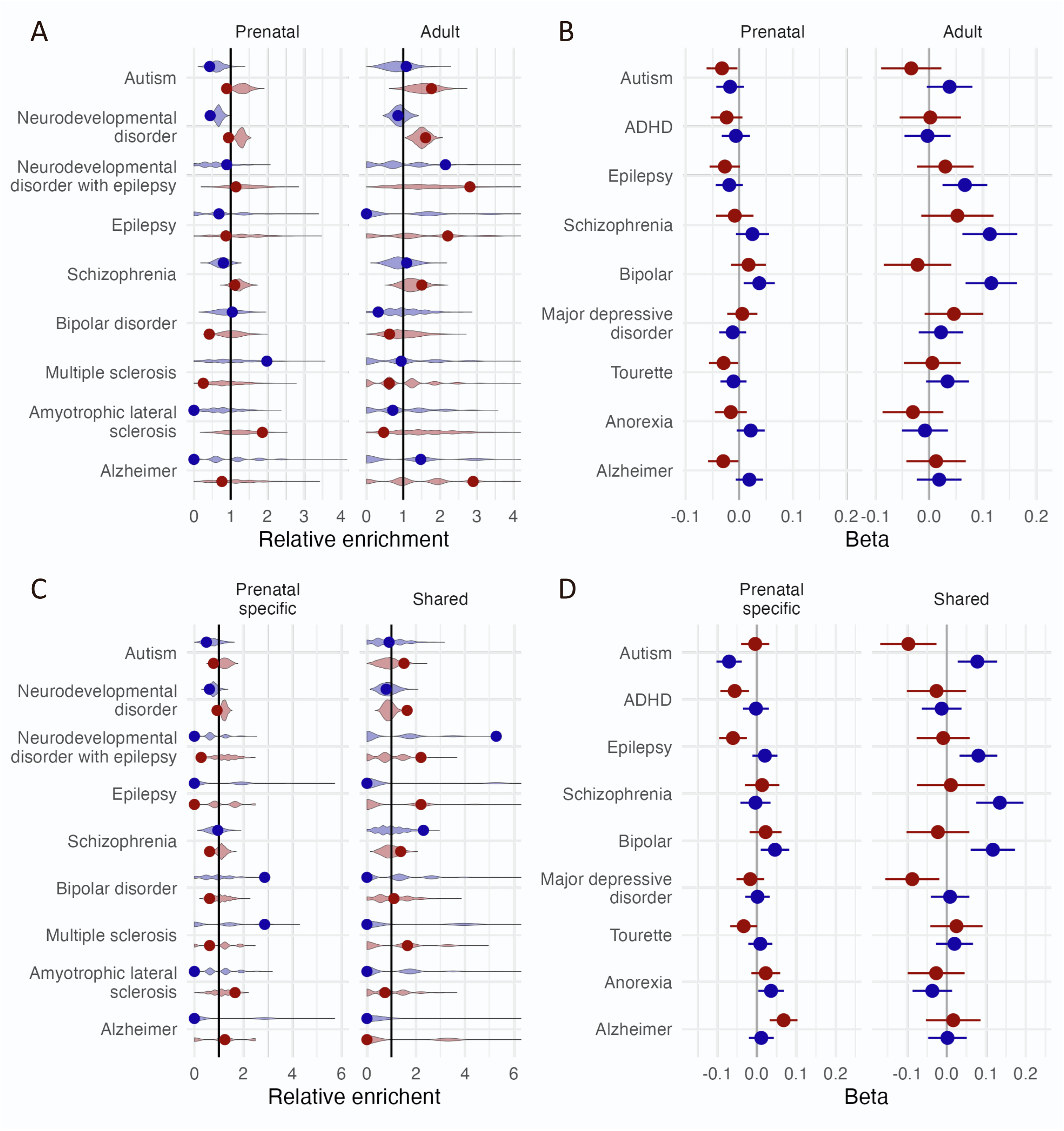

Figure S21: Enrichment analyses for the sex-DE genes in several neurodevelopmental and neurological diseases, related to Figure 4. A.) Enrichment analyses with hypergeometric test of gene lists from exome-sequencing studies in the prenatal and adult sex-DE genes. B.) Enrichment analyses with the MAGMA method of gene lists from GWAS in the prenatal and adult sex-DE genes. C.) Enrichment analyses with hypergeometric test of gene lists from exome-sequencing studies in the prenatal-specific and shared sex-DE genes. D.) Enrichment analyses with the MAGMA method of gene lists from GWAS in the prenatal-specific and shared sex-DE genes. The violinplots on the A.) and C.) plots represent the distribution of the relative enrichment for 1000 random gene lists and the point

denotes the enrichment observed for the true gene list. The error bars in B.) and D.) depict the SE of the beta values. No gene lists passed the significance threshold for both hypergeometric test (for the list of interest) and the permutation test (compared to the random gene sets) in A.) and C.) or the MAGMA P-value in B.) and D.), as defined in Methods.

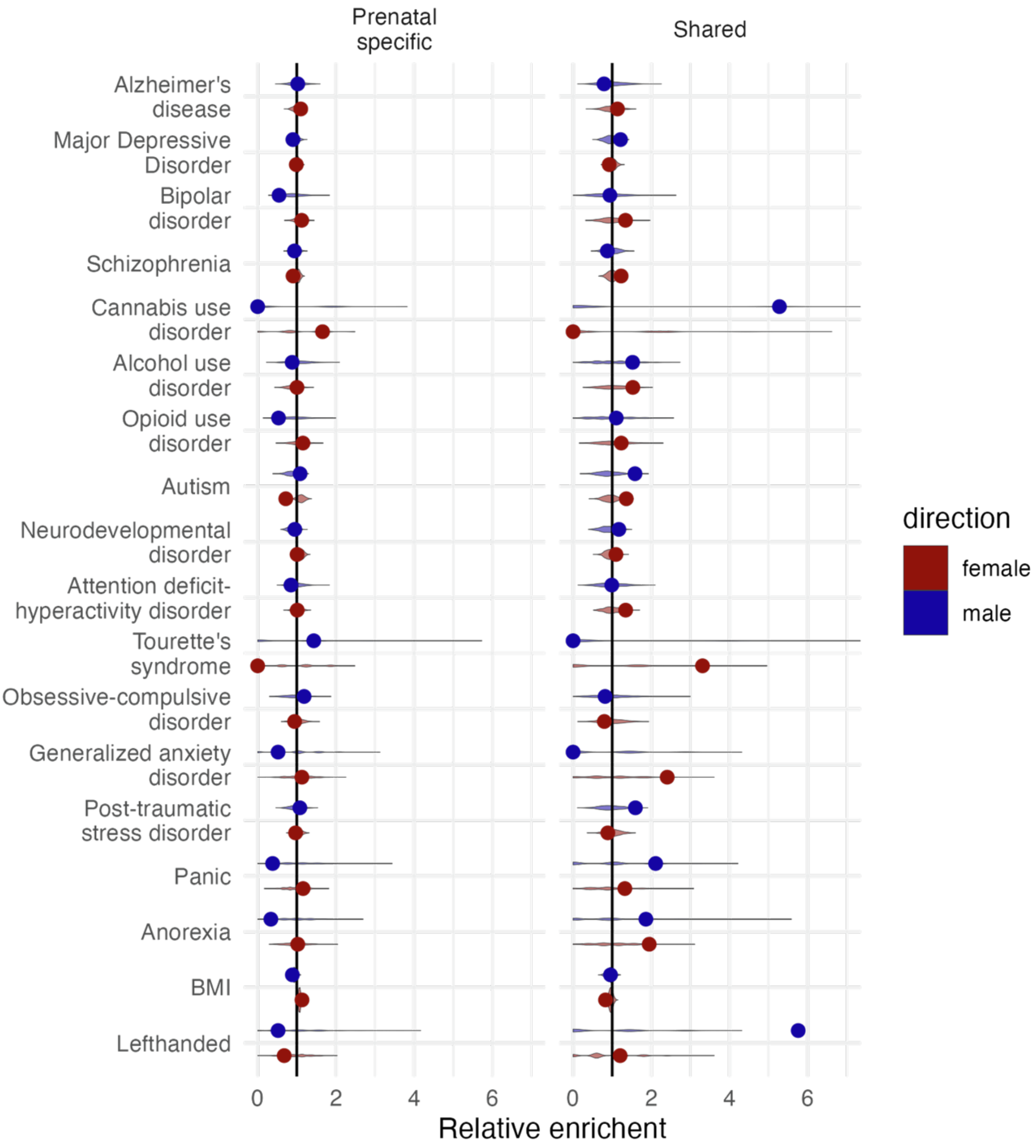

Figure S22: Enrichment analysis of 16 neuropsychiatric disorders and 2 control gene sets from Mulvey et al., 2024 study, related to Figure 4. Enrichments are assessed in prenatal specific and shared sex-DE genes (separated by direction of effect, i.e. female-biased and male-biased genes). The violinplots represent the distribution of the relative enrichment for the 1000 random gene lists and the point denotes the enrichment for the true gene list. No gene lists passed the significance threshold for both hypergeometric test (for the list of interest) and the permutation test (compared to the random gene sets).

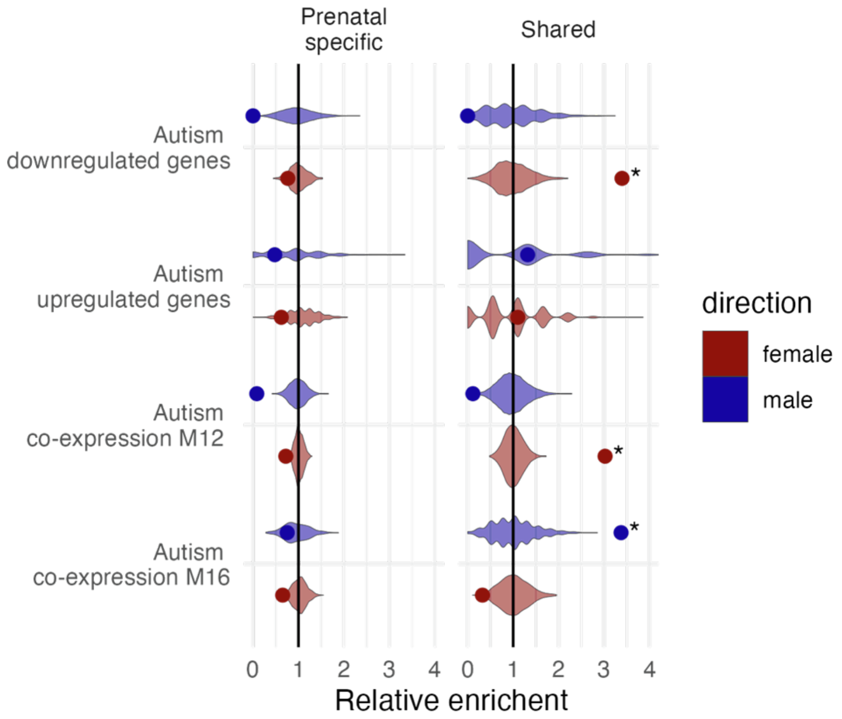

Figure S23: Enrichment analysis of case-control ASD associated genes, related to Figure 4. Enrichments are assessed in prenatal specific and shared sex-DE genes (separated by direction of effect, i.e. female-biased and male-biased genes). The violinplots represent the distribution of the relative enrichment for the 1000 random gene lists and the point denotes the enrichment for the true gene list. * indicates gene lists passing the significance threshold for both the hypergeometric test (for the list of interest) and the permutation test (compared to the random gene sets), as defined in Methods. The shared female-biased genes were enriched in genes downregulated in ASD cases as well as the ASD-associated co-expression module (M16) enriched for genes involved in immune and inflammatory responses. The shared male-biased genes were enriched for the co-expression module (M12) downregulated in ASD and enriched for genes with neuronal and synaptic functions. These enrichments were specific to shared genes and no enrichment for the prenatal-specific sex-DE genes was observed.

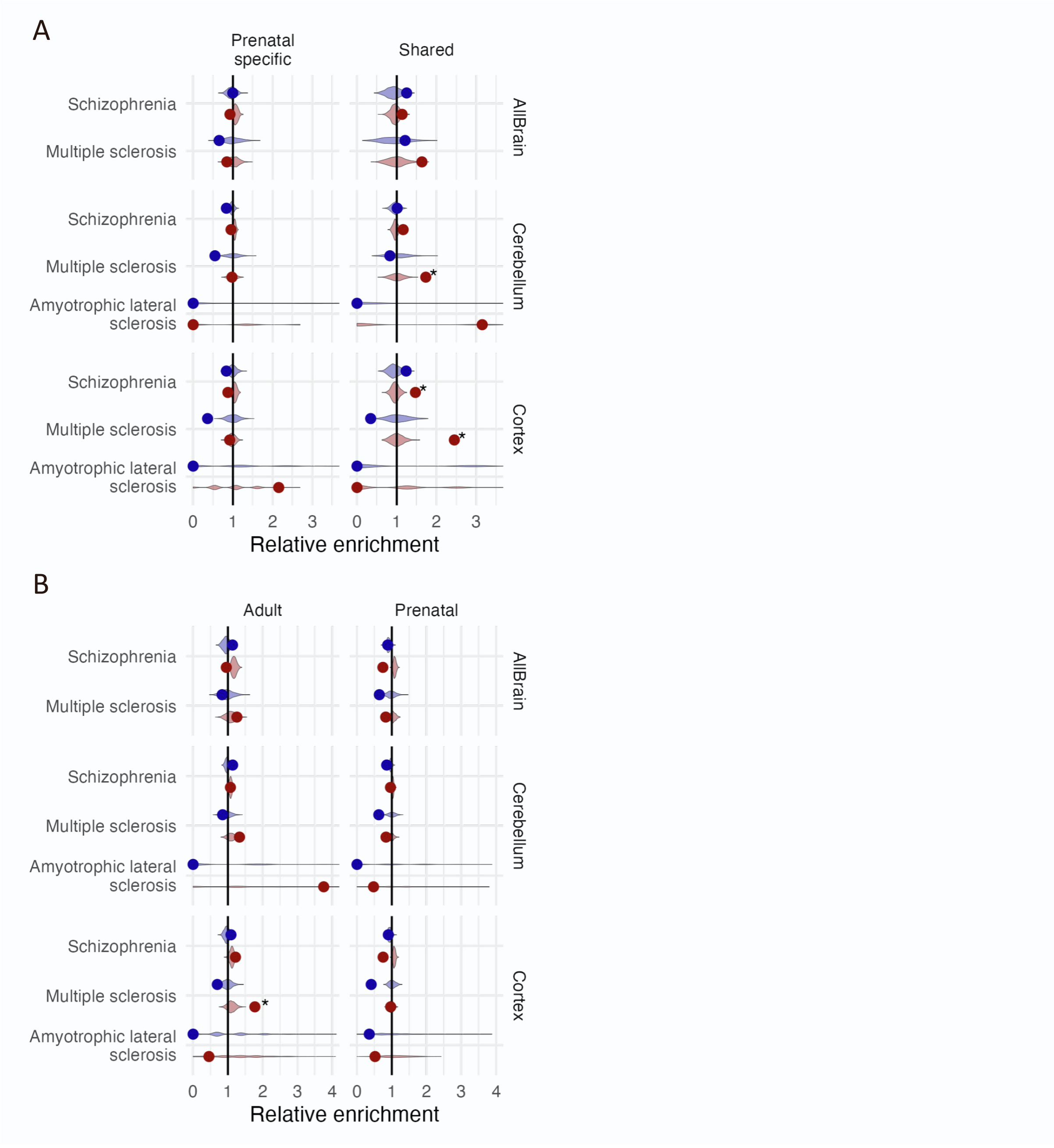

Figure S24: Enrichment analyses for the sex-DE genes in gene lists from co-regulation networks from the MetaBrain resource, related to Figure 4. A.) Enrichment analyses with the hypergeometric test in the prenatal-specific and shared sex-DE genes. B.) Enrichment analyses with the hypergeometric test in the prenatal and adult sex-DE genes. The violinplots on the A.) and B.) plots represent the distribution of the relative enrichment for the 1000 random gene lists and the point denotes the enrichment for the true gene list. * indicates gene lists passing the significance threshold for both the hypergeometric test (for the list of interest) and the permutation test (compared to the random gene sets), as defined in Methods.

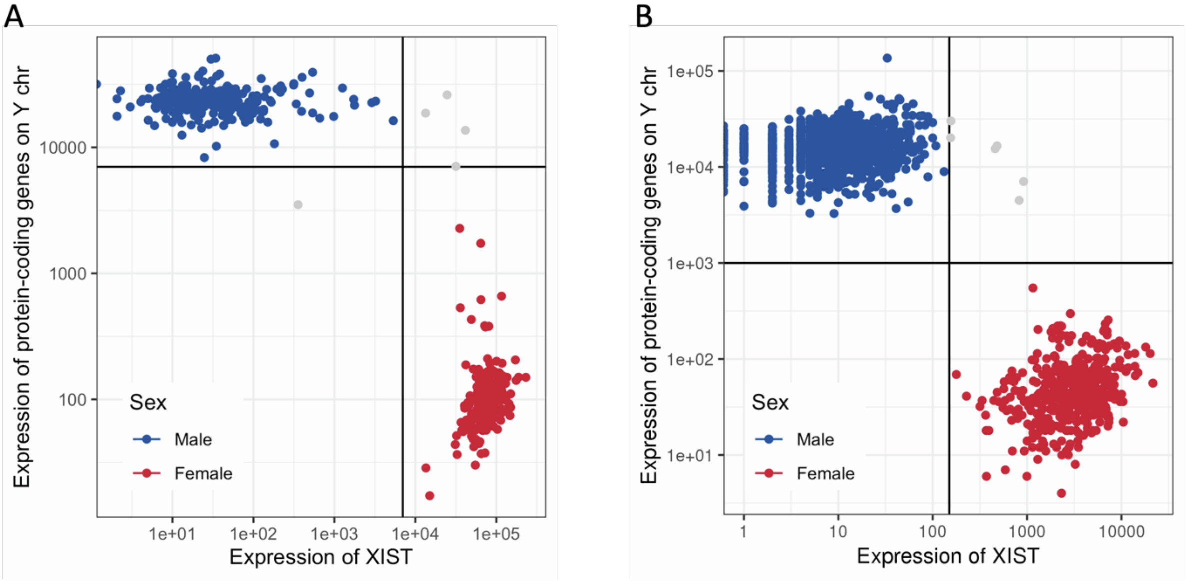

Figure S25: Sex labelling of the RNA-seq samples, related to STAR Methods. A.) Sex labels determined in the prenatal data. B.) Sex labels determined in the adult data. In both data sets the sample sex was determined using the expression of Y chromosome protein coding genes and XIST in the X chromosome.
